# Trajectories of Autism Symptom Severity Change During Early Childhood

**DOI:** 10.1007/s10803-020-04526-z

**Published:** 2020-05-14

**Authors:** Einat Waizbard-Bartov, Emilio Ferrer, Gregory S. Young, Brianna Heath, Sally Rogers, Christine Wu Nordahl, Marjorie Solomon, David G. Amaral

**Affiliations:** 1 Department of Psychology, University of California Davis, Davis, CA USA; 2 The MIND Institute and Department of Psychiatry and Behavioral Sciences, University of California Davis, 2825 50th Street, Sacramento, CA 95817 USA

**Keywords:** Autism spectrum disorder, Symptom severity, Early childhood, Sex differences

## Abstract

Autism symptom severity change was evaluated during early childhood in 125 children diagnosed with autism spectrum disorder (ASD). Children were assessed at approximately 3 and 6 years of age for autism symptom severity, IQ and adaptive functioning. Each child was assigned a change score, representing the difference between ADOS Calibrated Severity Scores (CSS) at the two ages. A Decreased Severity Group (28.8%) decreased by 2 or more points; a Stable Severity Group (54.4%) changed by 1 point or less; and an Increased Severity Group (16.8%) increased by 2 or more points. Girls tended to decrease in severity more than boys and increase in severity less than boys. There was no clear relationship between intervention history and membership in the groups.

Autism spectrum disorder (ASD) is a neurodevelopmental disorder characterized by deficits in social communication and social interaction, as well as restricted and repetitive behaviors (American Psychiatric Association 2013) and affects 1 out of every 59 children (Baio et al. 2018) in the United States. While the symptoms of ASD are commonly considered to be stable throughout life (Bieleninik et al. 2017), increasing evidence indicates that at least some individuals demonstrate substantial changes in the core features of ASD and/or comorbid conditions over time (Shattuck et al. 2007; Georgiades et al. 2014; Steinhausen et al. 2016; Hudry et al. 2018; Solomon et al. 2018). One example of substantial change is optimal outcome, defined as a decrease in autism symptoms in individuals previously diagnosed with ASD, so that they no longer meet diagnostic criteria (Fein et al. 2013).

Early indication of the potential for change was documented by Fountain et al. (2012) who described six distinct trajectories of symptom severity change using a very large cohort of participants (N = 6975). They described six developmental trajectories of social, communication and repetitive behavior functioning. While most children showed slow progress or little change, a small group (which they called Bloomers) demonstrated rapid gains between early childhood and adolescence. Although this study had the strength of involving a very large cohort, the conclusions were based on parental or provider reports obtained from the California Department of Developmental Services and not from direct clinical assessments. Change over time in autism symptoms is better assessed through clinical observations that are relatively free of the biases that are often inherent in parental report.

The Autism Diagnostic Observation Schedule (ADOS) has become the standard assessment instrument in the field of autism research (Lord et al. 2000). It is comprised of a series of structured and semi-structured tasks, allowing the trained examiner to evaluate a participant’s behavior, communication and social interaction, providing a standardized context for evaluation of autism symptom severity. The ADOS consists of several modules, each used with persons of a specific level of language development, ranging from pre-verbal to fluent speech. Different modules incorporate different tasks and demands and result in different scores, demonstrating the assessment’s strength in adapting to children’s varying language abilities but making it difficult to compare severity levels across individuals or across ages. The ability to reliably assess a child’s change in severity over time is a key concern for researchers, clinicians and parents alike (Shattuck et al. 2007; Magiati et al. 2014). To use the ADOS to assess changes in severity over time, Gotham et al. (2009) developed the ADOS Calibrated Severity Scores (CSS). To do so, they first created revised algorithms (Gotham et al. 2007) with the same number of items and similar content across modules that showed minimal association between the ADOS total scores, the child’s age and verbal IQ. Second, they used a sample of 1807 assessments from individuals diagnosed with ASD to create 18 age and language-based groups. Within each of the groups, percentiles were calculated for each of the ADOS diagnostic classifications (non-spectrum, ASD and autism). The calculation of percentages in the 18 developmental groups served as a basis for using the raw totals to produce a standardized, 10-point severity metric. This severity metric was found to have more uniform distributions across the developmental groups than raw scores and was less influenced by a participant’s characteristics (Gotham et al. 2009). The ADOS CSS has proven to be a better indicator of autism severity because it is relatively independent of verbal ability, age and other childhood characteristics. A number of studies have since employed and validated the CSS for this purpose (de Bildt et al. 2011; Hus Bal and Lord 2015; Messinger et al. 2015) and it has also been used to assess symptom severity change in large scale intervention studies (Estes et al. 2015; Pickles et al. 2016).

Gotham et al. (2012) were the first to use the ADOS CSS to examine autism severity trajectories in a sample of 345 children (63 girls and 282 boys), aged 2–15, each having completed between 2 and 8 assessments. Using generalized linear latent and mixed models statistics, they found that over 80% of participants could be assigned to a stable severity class, with two other small groups showing either an increase or a decrease in severity over time. Venker et al. (2014) explored autism severity trajectories using the ADOS CSS in a group of 129 children (17 girls and 112 boys) over four assessments between the ages of 2.5 and 5.5 years using latent class growth models. Their findings are consistent with Gotham et al. (2012). They identified the same trajectory classes with almost 80% of children showing stable levels of severity over time. In 2015, Szatmari et al. used a semiparametric, group-based approach to study a sample of 421 2–6-year-old children (66 girls and 355 boys) who were assessed at three time points, also using the ADOS CSS. They observed two severity trajectories; a large group (88.6%) characterized with higher initial severity that demonstrated stable severity across time and a small group (11.4%) with initially low severity and decreases over time. Kim et al. (2016) identified subgroups based on autism symptoms and other aspects of clinical profiles and short term outcomes using hierarchical clustering analysis. Their sample included 100 toddlers (16 girls and 84 boys) evaluated in the second and third years of life using the ADOS CSS. Their results indicated that autism symptom severity remained stable over a 1 year period for 84% of their participants while 16% demonstrated an increase in severity.

In 2017, Clark et al. reported on a group of 48 children (12 girls and 36 boys) evaluated for ASD symptoms across three time points using the ADOS CSS, from age 2 to 9 years of age. They divided the sample into groups based on diagnostic stability, producing a non-stable ASD group (13 children) comprised of participants who, as they grew older, no longer met diagnostic criteria and an ASD stable group (35 children), comprised of participants who retained diagnosis over time. Both groups showed significant autism symptom severity change over time. Analysis based on simple main effects demonstrated that the non-stable ASD group consistently decreased in their autism severity over time while the ASD stable group decreased in severity during preschool age but then increased again during school age, to return to their toddlerhood levels. In 2018, Kim et al. identified variability in autism symptom trajectories of 149 toddlers (30 girls and 119 boys), 14–36 months old, referred for autism evaluation. Using latent class growth analysis of the ADOS CSS, they identified four groups: a non-spectrum group (25%); a worsening group (27%) with initially low severity levels that increased over time; a moderately-improving group (25%) that showed a slight decrease in severity and a severely affected group (23%) that maintained high severity levels over time. Recently, Pellicano et al. (2019) evaluated autism symptom severity across a 9 year period, based on two assessments, using the ADOS CSS in a sample of 27 individuals (2 girls and 25 boys). Participants ranged from 8 to 11 years at initial assessment and 16 to 20 years at second assessment. While group mean severity level remained stable over time, there was high variability in individual participant’s symptom trajectories. Reliable change in severity levels was identified for more than half the sample: 29% of participants increased in severity over time, 29% decreased in severity and 42% remained stable.

Intervention studies have also demonstrated the ability of treatment to impact and reduce symptom severity levels. The Pre-school Autism Communication Trial (PACT), a parent-mediated social communication intervention targeting autism symptoms, was administered to 152, 2–4-year-old children, autism severity was measured using ADOS CSS and analysis was done using mixed-effect ordinal logistic regression. PACT was shown to successfully reduce autism symptom severity at treatment end point, an effect which remained at follow up assessment almost 6 years later (Pickles et al. 2016). Giserman-Kiss and Carter (2019) evaluated autism symptoms following intervention for 60 children (8 girls and 52 boys) of diverse backgrounds using two time points; at initial assessment (age 19–34 months) and at follow up assessment (42–70 months) after having received intervention in the community. Paired t-test analysis showed that, on average, children demonstrated significant decreases in symptom severity between initial and follow up assessments. Thus, while earlier studies using the ADOS CSS had emphasized relative stability of autism symptoms for most individuals with small groups either decreasing or increasing with time, more recent studies have highlighted variability in symptom trajectories and a higher proportion of change in severity levels than previously depicted.

In the current study, we used the ADOS CSS to explore changes in autism symptom severity during early childhood i.e. for children between 3 and 6 years of age. Focusing on autism severity changes in early childhood is important for a variety of reasons. First, early childhood is a period of substantial brain growth with the potential for enormous plasticity (Cao et al. 2017; Walhovd et al. 2017; Gilmore et al. 2018; Oldham and Fornito 2018; Lebel et al. 2019). Second, because early childhood is the usual time of initial diagnosis, it has become the primary target age for early intervention (Rogers and Dawson 2010). While previous studies of autism severity have also included early childhood participants, (Venker et al. 2014; Szatmari et al. 2015), the current study has a number of unique strengths. First, this is a single site study; participants were recruited through the Autism Phenome Project (APP), a multidisciplinary longitudinal project in its 14th year at the MIND (Medical Investigation of Neurodevelopmental Disorders) Institute of the University of California, Davis. Participation in the APP includes a comprehensive assessment battery starting when children are 2–3.5 years of age. Thus, the participants’ age range at baseline is narrow. Second, and germane to the assessment of symptom severity over time, all clinical evaluations are carried out consistently at the same location (the MIND Institute) by licensed psychologists trained to research standards and under the supervision of the same clinical team. Third, a comprehensive database of information is available for all participants including biological data (such as magnetic resonance imaging), medical records, cognitive and language measures and intervention history. Fourth, increased representation of girls in the cohort enabled evaluation of sex differences in symptom severity change.

The overarching aim in this study was to evaluate trajectories of symptom severity across early childhood and to investigate what associated factors might be influences. We focused on two questions: (1) Does the severity of autism symptoms change in individual children across early childhood and (2) Was the amount or direction of change affected by initial severity levels, intervention intensity, sex, IQ or level of adaptive functioning.

## Methods

### Participants

Participants were enrolled in the University of California (UC) Davis MIND Institute Autism Phenome Project or Girls with Autism Imaging of Neurodevelopment Study (GAIN). Participants enrolled between 2 and 3.5 years of age. The study protocol includes a comprehensive assessment battery, collecting neuropsychological, medical, behavioral and biological information. The present study reports behavioral data related to autism symptom severity, cognitive function and adaptive behavior collected at Time 1, the baseline assessment, and Time 3, which served as the follow up assessment. Time 2 (1 year following Time 1) is not addressed in the current study since only magnetic resonance imaging data were collected at that time point. Nonetheless, we maintain a consistent timing nomenclature across all publications. The study was approved by the UC Davis Institutional Review Board and informed consent was obtained from the parent or guardian of each participant.

One hundred and twenty-five participants were evaluated, 89 boys and 36 girls. Participant characteristics are provided in Table 1. Inclusion criteria were based on the NIH Collaborative Programs of Excellence in Autism. Participants had received a community diagnosis of ASD that was confirmed by a licensed clinician at the MIND Institute using the ADOS-2 and the Autism Diagnostic Interview-revised (ADI-R) (Lord et al. 1994, 2000). A diagnosis was confirmed if they met the ADOS-2 cut off score for either autism or ASD and exceeded the ADI-R cut off score for autism on either the Social or Communication subscales while being within two points of this criterion on the other subscale. Study participants were required to be English speaking, reside with at least one biological parent, be ambulatory and not diagnosed with any severe motor, vision, hearing or other chronic health issues that might hinder participation.

**Table 1:** Demographic information

		Time 1			Time 3		
		All	Boys	Girls	All	Boys	Girls
N (%)		125, 100%	89, 71.2%	36, 28.8%	125, 100%	89, 71.2%	36, 28.8%
Age (months)	${\overline{\text{x}}}$ (SD)	35.54 (5.58)	34.56 (5.48)	37.97 (5.14)	68.31 (10.90)	67.98 (11.71)	69.14 (8.87)
ADOS CSS	${\overline{\text{x}}}$ (SD)	7.30 (1.71)	7.37 (1.72)	7.14 (1.69)	7.00 (2.14)	7.27 (2.02)	6.36 (2.32)
Intervention:							
Hours	${\overline{\text{x}}}$ (SD)	882 (819)	925 (888)	783 (631)	3218 (1848)	3233 (1937)	3180 (1618)
Intensity	${\overline{\text{x}}}$ (SD)	2353 (712)	2385 (772)	2280 (556)	3790 (1652)	3824 (1715)	3696 (1487)
IQ	${\overline{\text{x}}}$ (SD)	66.59 (21.18)	65.33 (20.81)	69.79 (22.80)	79.07 (31.45)	77.80 (31.24)	82.22 (32.20)
VABS-II:							
Composite score	x̅ (SD)	76.73 (11.21)	78.42 (11.10)	72.59 (10.20)	76.61 (16.35)	76.61 (16.10)	76.61 (17.22)
Motor skills	${\overline{\text{x}}}$ (SD)	87.38 (13.71)	89.10 (13.39)	83.18 (13.00)	81.54 (14.85)	81.24 (15.66)	82.31 (12.85)
Socialization	${\overline{\text{x}}}$ (SD)	75.17 (11.80)	76.98 (12.00)	70.71 (10.00)	74.38 (18.65)	74.78 (18.50)	73.42 (19.27)
Communication	${\overline{\text{x}}}$ (SD)	75.14 (15.32)	76.11 (14.74)	72.74 (16.50)	81.25 (19.67)	80.66 (19.47)	82.67 (20.36)
Daily living skills	x̅ (SD)	80.11 (12.44)	81.76 (12.29)	76.03 (12.00)	78.81 (17.80)	79.35 (17.09)	77.51 (19.62)

### Measures

We used measures common for assessment of autism symptom severity, cognitive abilities (IQ) and adaptive functioning in children within this age range. These included the Autism Diagnostic Observation Schedule-2: ADOS-2 (Lord et al. 2000), Mullen Scales of Early Learning: MSEL (Mullen 1995), Differential Abilities Scales-II: DAS-II (Elliot 2007), and the Vineland Adaptive Behavioral Scales: VABS II (Sparrow et al. 2005). All assessments were either conducted or supervised by a trained, licensed clinical psychologist who specializes in ASD and who had reached research reliability for these instruments. To increase the likelihood of successful testing and to allow children to demonstrate their full abilities, several accommodations were put in place as part of the assessment procedure. For example, children were given as many breaks as needed to use the bathroom, eat snacks or simply rest. If a child experienced distress at any time, they received a break to rest and gather themselves with the help of their parents. To increase motivation for participation, methods such as sticker charts were used. Testing children as young as these is always challenging. However, the probability of accurate testing was increased since these procedures were carried out by experts in child development. Assessment measures included the following:

#### Autism Diagnostic Observation Schedule-2: ADOS-2 (Lord et al. 2000)

The ADOS-2 is a semi-structured, standardized assessment instrument considered to be the gold standard for ASD diagnosis. The ADOS-2 includes five modules increasing in difficulty which are assigned based on a participant’s language development and age. The Calibrated Severity Score (Gotham et al. 2009) provides a quantitative assessment of increasing severity of autism related symptoms (1–2: “minimal-to-no evidence”, 3–4: “low”, 5–7: “moderate” and 8–10: “high”), with a score of 4 or above meeting criteria for an ASD diagnosis. All clinical evaluations were carried out consistently at the same location (the UC Davis MIND Institute) by licensed psychologists trained to research standards and under the supervision of the same clinical team. The MIND Institute procedure for ensuring research reliability of clinicians administering the ADOS is adapted from the procedure required by the developers of the ADOS-2. Research reliability is established for all ADOS modules by reaching agreement of 80% or higher (i.e. reliability of 0.80) with a research-reliable clinician on three consecutive ADOS assessments for each module set (Set 1: Modules Toddler, 1 and 2; set 2: Modules 3 and 4). Once reliability is achieved, administrators take part in regular clinical supervision sessions of ADOS administration and coding, facilitated by a certified ADOS trainer. Random double coding was employed using both live assessments and video recordings. A second rater was regularly employed in the case of a child previously meeting criteria for diagnosis but failing to meet at a later assessment or in cases that the first rater felt another professional opinion was warranted.

#### Mullen Scales of Early Learning: MSEL (Mullen 1995)

The MSEL is a standardized assessment tool which measures cognitive and developmental functioning of children up to 68 months of age. At Time 1, verbal, nonverbal and combined IQ were estimated by calculating ratio developmental quotient scores, dividing average verbal, nonverbal and combined MSEL subscale age equivalents by chronological age. However, due to a substantial proportion of participants achieving the lowest possible standard score, a ratio developmental quotient was calculated (mental age/chronological age * 100) to provide more specific individual estimates of nonverbal, verbal and combined IQ.

#### Differential Abilities Scales-II (DAS-II) (Elliot 2007)

The DAS-II is a standardized measure that assesses children’s cognitive abilities between the ages of 2.5 and 17 years. Participants completed the core battery of either the DAS-II Upper Early Years or the School Age forms. Participants who were not able to achieve basal scores on the DAS-II at Time 3 were administered the MSEL. Developmental quotients (DQ) were used to calculate verbal, nonverbal and combined IQ scores.

#### Vineland Adaptive Behavior Scales, Second Edition: VABS II, Parent / Caregiver Rating Form (Sparrow et al. 2005)

The VABS II measures adaptive function from birth to adulthood. It yields a standardized composite score, percentile ranks and adaptive levels. The current study analyzed the standardized composite score and four of the domains: Motor Skills, Socialization, Communication and Daily Living Skills, using parents’ assessment of their child’s behavior.

#### Services, Treatment and Intervention Data

At each visit, the child’s caregiver(s) completed a form inquiring about current and previous intervention received by the child, including information regarding type and duration of treatment. This form was adapted from the Collaborative Programs of Excellence in Autism. An intensity score for intervention was calculated based on the following formula: (weeks of intervention * hours per week * number of adults / number of children present).

### Data Analysis

**Fig. 1:**
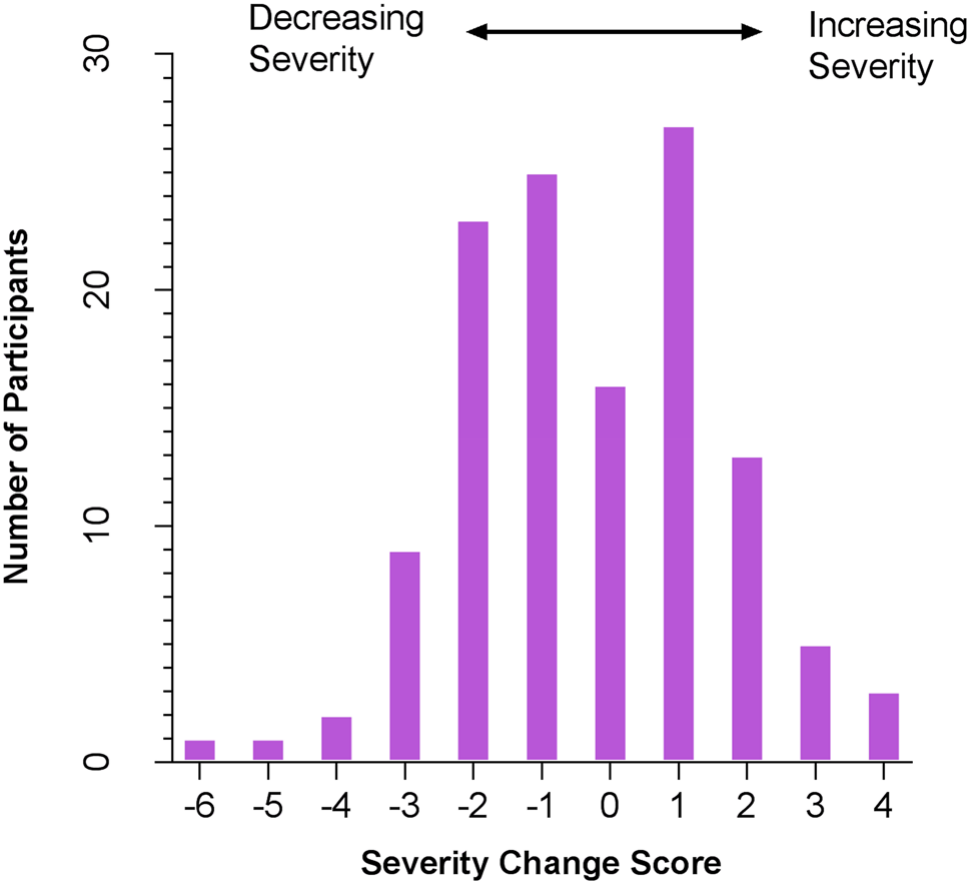
Distribution of change scores in the sample. Decreases in autism severity from Time 1 to Time 3 are indicated as negative numbers whereas increases in autism severity are indicated as positive numbers

To evaluate the profiles of individual change demonstrated by the participants across time, a severity change score was computed for each participant (Time 3 ADOS CSS–Time 1 ADOS CSS) (Fig. 1). The mean severity change score for the sample was -0.30 (SD: 1.91) and the distribution ranged from − 6 (decrease of 6 points in symptom severity over time) to + 4 (increase of 4 points in symptom severity over time).

We sought to explore the characteristics of children who increased, decreased or remained stable in autism severity. To determine how much change from Time 1 to Time 3 is meaningful, we used the Reliable Change Index statistic (RCI; Jacobson and Truax 1991)

$$RCI_{{Z\,SCORE}} = \frac{{({\text{ADOS}}\,{\text{CSS}}{\mkern 1mu} {\text{Time}}{\mkern 1mu} 3 - {\text{ADOS}}\,{\text{CSS}}{\mkern 1mu} {\text{Time}}{\mkern 1mu} 1)}}{{\sqrt {2\left( {SD\sqrt {1 - r_{{xy}} } } \right)^{2} } }}$$

The RCI indicates what amount of change in clinical data can be considered statistically significant (Anderson et al. 2014; de Souza Costa and de Paula 2015; Hudry et al. 2018; Pellicano et al. 2019). The RCI was calculated using the means of CSS at Time 1 (7.30) and Time 3 (7.00), the standard deviation (SD) at Time 1 (1.71) and the reliability of the ADOS CSS, which was assumed to be 0.80 (as this is the minimal value for test–retest reliability). All analyses were performed in R version 3.5.1 (R_Core_Team. 2018).

## Results

### Defining Three Groups based on Change in Severity

The RCI was computed for the entire sample (Brown et al. 2015), yielding a value of 2.12 (or 2.0 rounded to the nearest integer). Thus, by this measure, a change of 2 points or more can be considered to be a significant change over time. Based on these results, we created three groups of severity change: a Decreased Severity Group (DSG) comprised of participants who had a decrease in their ADOS CSS score of 2 or more points from Time 1 to Time 3; a Stable Severity Group (SSG) who had a change of severity score of 1 point or less; and an Increased Severity Group (ISG) comprised of participants with an increase in CSS of 2 or more points (see Table 2).

**Table 2:** Demographic information and descriptive statistics for the three groups

		DSG	SSG	ISG
N (%)		36 (28.8%)	68 (54.4%)	21 (16.8%)
Sex	Boys (N = 89, 71.2%)	23 (25.8%^a^)	48 (53.9%)	18 (20.2%)
	Girls (N = 36, 28.8%)	13 (36.1%)	20 (55.6%)	3 (8.3%)
Age (months)	Time 1: ${\overline{\text{x}}}$ (SD)	34.9 (5.4)	35.8 (5.9)	35.9 (5.0)
	Time 3: ${\overline{\text{x}}}$ (SD)	67.5 (10.1)	69.3 (12.0)	66.3 (8.8)
ADOS CSS	Time 1: ${\overline{\text{x}}}$ (SD):			
	All	7.82 (1.84)	7.35 (1.61)	6.19 (1.17)
	Boys	8.09 (2.00)	7.48 (1.52)	6.17 (1.25)
	Girls	7.38 (1.66)	7.10 (1.83)	6.33 (0.58)
	Time 3: ${\overline{\text{x}}}$ (SD):			
	All	5.27 (2.15)	7.4 (1.71)	8.71 (1.23)
	Boys	5.61 (1.95)	7.52 (1.74)	8.72 (1.27)
	Girls	4.69 (2.43)	7.10 (1.65)	8.67 (1.15)
Change score	x̅ (SD)	− 2.55 (0.94)	0.03 (0.88)	2.52 (0.75)
Intervention history	Total hours:			
	Time 1: ${\overline{\text{x}}}$ (SD)	961 (909)	898 (854)	720 (535)
	Time 3: ${\overline{\text{x}}}$ (SD)	2851 (1610)	3415 (1910)	3213 (2026)
	Intensity:			
	Time 1: ${\overline{\text{x}}}$ (SD)	2430 (766)	2340 (748)	2284 (503)
	Time 3: ${\overline{\text{x}}}$ (SD)	3332 (1283)	4028 (1627)	3821 (2170)
IQ	Time 1: ${\overline{\text{x}}}$ (SD)	71.28 (22.95)	66.28 (21.86)	59.53 (12.92)
	Time 3: ${\overline{\text{x}}}$ (SD)	88.50 (30.19)	76.77 (31.76)	70.35 (30.03)
VABS-II	Composite score:			
	Time 1: ${\overline{\text{x}}}$ (SD)	76.97 (10.46)	76.79 (11.55)	76.05 (11.93)
	Time 3: ${\overline{\text{x}}}$ (SD)	81.29 (16.87)	75.36 (16.28)	70.93 (13.01)
	Motor skills:			
	Time 1: ${\overline{\text{x}}}$ (SD)	86.34 (14.21)	88.02 (13.73)	87.16 (13.33)
	Time 3: ${\overline{\text{x}}}$ (SD)	85.52 (13.77)	80.16 (16.20)	78.29 (9.50)
	Socialization:			
	Time 1: ${\overline{\text{x}}}$ (SD)	74.83 (9.86)	75.64 (12.87)	74.21 (11.81)
	Time 3: ${\overline{\text{x}}}$ (SD)	77.26 (20.78)	74.27 (17.95)	68.13 (15.65)
	Communication:			
	Time 1: ${\overline{\text{x}}}$ (SD)	76.80 (13.66)	74.67 (16.50)	73.63 (14.56)
	Time 3: ${\overline{\text{x}}}$ (SD)	86.80 (20.14)	79.76 (19.43)	74.53 (17.36)
	Daily living skills:			
	Time 1: ${\overline{\text{x}}}$ (SD)	81.71 (13.74)	79.08 (11.2)	80.63 (14.15)
	Time 3: ${\overline{\text{x}}}$ (SD)	84.74 (17.77)	76.62 (17.3)	74.2 (17.54)

^a^Percentages out of each sex

The largest group of children (54.4% of the sample) showed stable severity over time (stable severity group—SSG). The second largest group of participants (28.8% of the sample) decreased in severity over time (decreased severity group—DSG) and the smallest group (16.8% of the sample) increased in severity over time by 2 or more points (increased severity group—ISG) (Table 2). An ANOVA of change scores indicated that all groups were different from each other (F(2,122) = 232.14, *p* < 0.001, Eta squared 0.79, Tukey test—all comparisons p < 0.001).

To examine possible factors affecting differences between the groups, we considered three variables: participant’s age, level of cooperation during the ADOS (based on clinician observations during the administration of the ADOS and measured through the three ADOS items concerning Other Abnormal Behaviors), and type of ADOS module administered. Age did not differ across the three groups (Table 2) at either Time 1 (F(2,122) = 0.38, *p* = 0.69) or Time 3 (F(2,122) = 0.72, *p* = 0.49). Child's level of cooperation during assessments did not differ across the three groups either: Overactivity/Agitation (Time 1: F(2,118) = 0.99, *p* = 0.37, Time 3: F(2,87) = 0.74, *p* = 0.48), Tantrums, Aggression, Negative or Disruptive Behavior (Time 1: F(2,118) = 0.30, *p* = 0.74, Time 3: F(2,87) = 0.64, *p* = 0.53) or Anxiety (Time 1: F(2,117) = 0.16, *p* = 0.85, Time 3: (F(2,87) = 1.74, *p* = 0.18). Regarding the ADOS module, at Time 1 most participants were administered module 1 (DSG: N = 28, 77.8%; SSG: N = 55, 80.9%; ISG: N = 19, 90.5%), with fewer participants administered module 2 (DSG: N = 8, 22.2%; SSG: N = 13, 19.1%; ISG: N = 2, 9.5%) and none administered module 3. At Time 1, there were no significant differences in proportions of modules administered across the three severity change groups (*X*^2^(2) = 1.48, *p* = 0.48). At Time 3, the DSG had a higher proportion of children tested with module 3 (N = 17, 47.2%) and almost equal proportions of module 1 (N = 10, 27.8%) and module 2 (N = 9, 25%). This indicates that the DSG did not decrease in severity because its participants were being tested with a less demanding module. The SSG had similar proportions of each module (module 1: N = 20, 29.4%; module 2: N = 23, 35.3%; module 3: N = 25, 36.8%) and the ISG showed a higher proportion of module 1 (N = 9, 42.9%), followed by module 2 (N = 7, 33.3%) and module 3 (N = 5, 23.8%). There was no difference between groups (*X*^2^(4) = 3.7, *p* = 0.45) in modules administered at Time 3. Thus, neither participant’s age, level of cooperation during the ADOS, nor type of ADOS module administered influenced the composition of the severity groups.

### Initial Average Severity Levels for the Three Groups

**Fig. 2:**
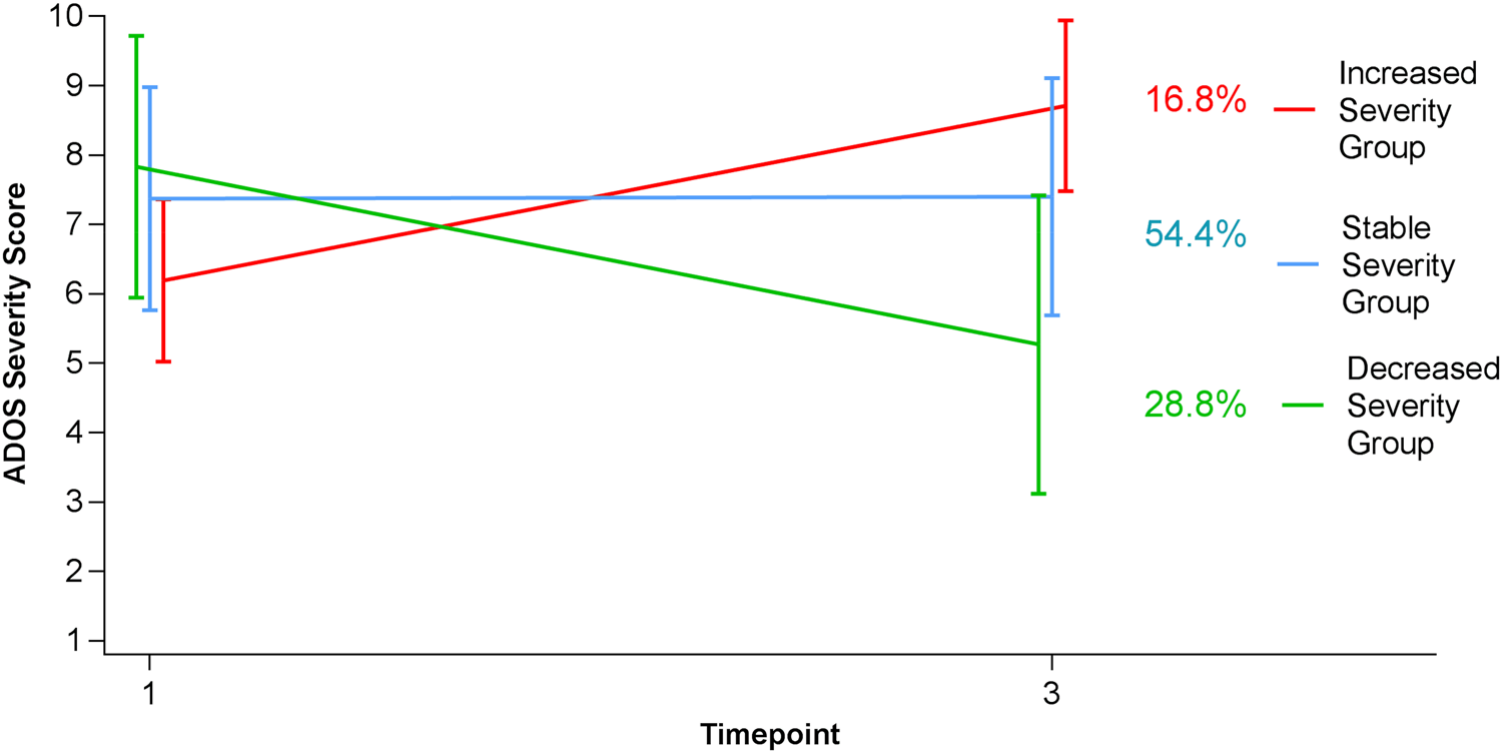
Group severity trajectories based on group ADOS CSS means at Time 1 and Time 3

Assignment of participants to groups was based on their change in autism severity over time, regardless of their initial severity scores. To better understand the patterns of severity at the two time points, severity means for the three groups were examined (Fig. 2). An ANOVA of initial severity scores at Time 1 showed significant differences between the groups (F(2,122) = 6.81, *p* = 0.001, Eta squared = 0.10). The ISG had a lower severity score compared to both the SSG (*p* < 0.001, Cohen’s d = 0.77) and DSG (*p* < 0.001, Cohen’s d = 0.99) who did not differ from each other (*p* = 0.21). The ISG’s Time 1 severity level was also significantly lower than the sample’s overall mean for initial severity (*p* < 0.001, Cohen’s d = 0.68). The DSG and SSG, however, did not differ from the sample’s general mean (DSG: *p* = 0.14, SSG: *p* = 0.80) for initial severity level. We also found that severity level at Time 1 was negatively related to the change score (r = -0.31, *p* < 0.001) i.e., a lower Time 1 severity score was associated with a more positive change score (greater increase in severity). Mean group severity levels were also different at Time 3 (F(2,122) = 28.19, *p* < 0.001, Eta squared = 0.40). At time 3, the ISG had the highest severity score compared to both of the other groups (ISG-SSG: *p* = 0.01, Cohen’s d = 0.82; ISG-DSG: *p* < 0.001, Cohen’s d = 1.84), the DSG had the lowest severity score (DSG-SSG: *p* < 0.001, Cohen’s d = 1.13), and the SSG showed an intermediate severity level.

**Fig. 3:**
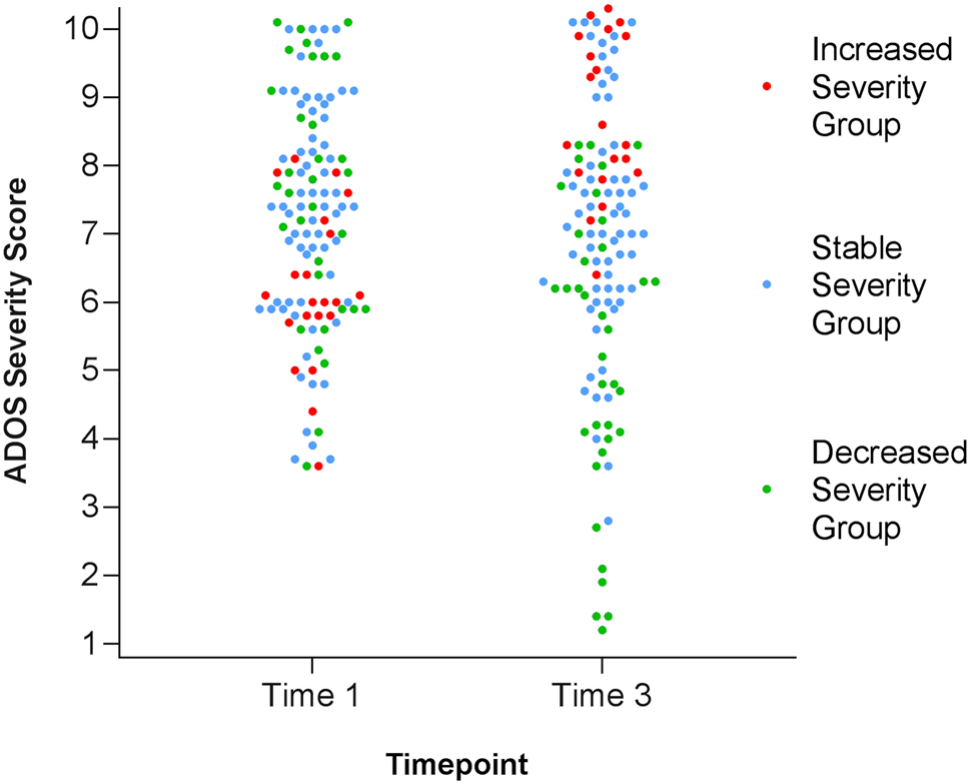
Scatterplot of individual ADOS CSS of all children in the sample at Time 1 and Time 3, by group membership. The DSG and SSG show a large range of individual severity scores at both Time 1 and Time 3 while The ISG shows a narrower range. Note, scores at Time 1 are plotted with jitter so that all individuals can be seen; participants plotted slightly below 4 actually received an ADOS CSS of 4

On average, the group of children who increased in severity from Time 1 to Time 3 had the lowest severity level at the first time point. In comparison, the groups of children who experienced either a decrease in severity or had stable severity levels had higher severity levels at Time 1 than the ISG. Both the DSG and SSG demonstrated similarly large ranges of initial severity scores (*p* = 0.29) and their group means were not different from each other (*p* = 0.33) (Fig. 3). While all groups were comprised of some participants with low initial severity levels, 71.4% of children in the ISG had a CSS of 6 or under at Time 1 compared to 27.8% in the DSG and 27.9% in the SSG.

### Intervention History for the Three Groups

Intervention history differences (total number of intervention hours received and intensity of intervention based on duration and number of hours per week) were evaluated for the three groups (see Table 2). ANOVA of total number of intervention hours did not show a significant difference between the groups at either Time 1 (F[2,114] = 1.04, *p* = 0.57) or Time 3 (F[2,114] = 0.56, *p* = 0.36). Similarly, ANOVA of intensity of intervention did not show significant group differences either at Time 1 (F[2,112] = 0.27, *p* = 0.76) or Time 3 (F[2,113] = 2, *p* = 0.14).

### Sex Differences in Severity Change over Time

The mean severity level of girls at Time 1 was not significantly different from that of boys (t(65.82) = -0.69, *p* = 0.49). However, at Time 3, girls had, on average, significantly lower severity scores compared to boys (t(57.51) = -2.06, *p* = 0.04, Cohen’s d = 0.43). This difference in the way each sex changed in severity from Time 1 to Time 3 was not related to IQ since there were no differences between the sexes in IQ at either Time 1 (t(59.12) = 1.03, *p* = 0.31) or Time 3 (t(63.09) = 0.70, *p* = 0.49).

**Fig. 4:**
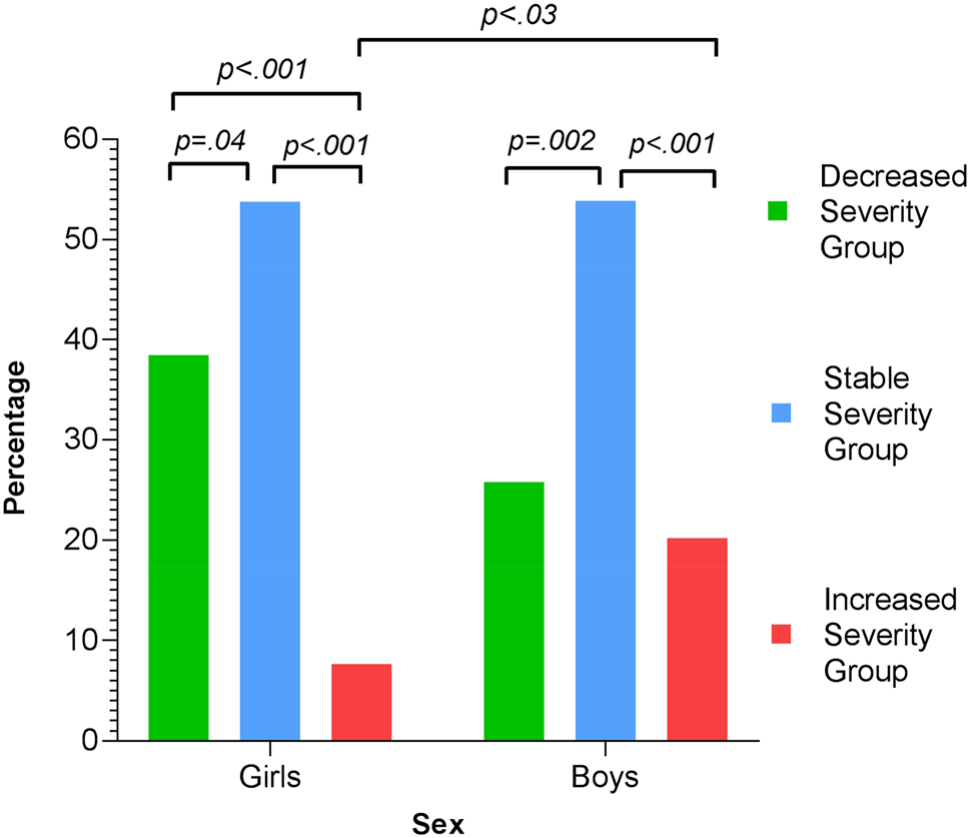
Percentages of girls and boys in the three groups. There was no significant difference between the proportions of boys in the DSG (25.8%) and the ISG (20.2%). However, there was a significant difference between the proportions of girls in the DSG (36.1%) and the ISG (8.3%)

The proportions of boys and girls across each of the three groups was different (boys -*X*^2^(2) = 17.42, *p* < 0.001, and girls -*X*^2^(2) = 12.17, *p* = 0.002) (Table 2; Fig. 4). The proportion of boys in the SSG (N = 48, 53.9%) was larger than in the other groups (SSG-DSG: *X*^2^(1) = 9.91, *p* = 0.002; SSG-ISG: *X*^2^(1) = 15.33, *p* < 0.001*)*. The proportions of boys who either decreased or increased in severity were similar (DSG, N = 23, 25.8%; ISG, N = 18, 20.2%), (*X*^2^(1) = 0.68, *p* = 0.41). The proportion of girls who demonstrated stable severity was similar to the boys and different from the other two groups (SSG: N = 20, 55.6%), (SSG-ISG: *X*^2^(1) = 34.95, *p* < 0.001; SSG-DSG: *X*^2^(1) = 4.14, *p* = 0.04). However, there was a different profile of girls who experienced change in severity compared to the boys. There was a higher proportion of the girls that decreased in severity (DSG) 36.1% (N = 13) than increased 8.3% (N = 3) (DSG-ISG: *X*^2^(1) = 17.37, *p* < 0.001). The proportion of girls in the ISG was lower than the proportions of girls in both of the other groups, as well as the proportion of boys in the ISG (*X*^2^(1) = 4.94, *p* = 0.03). In other words, the girls were over-represented in the DSG and under-represented in the ISG (Table 2; Fig. 4).

### IQ for the Three Groups

**Fig. 5:**
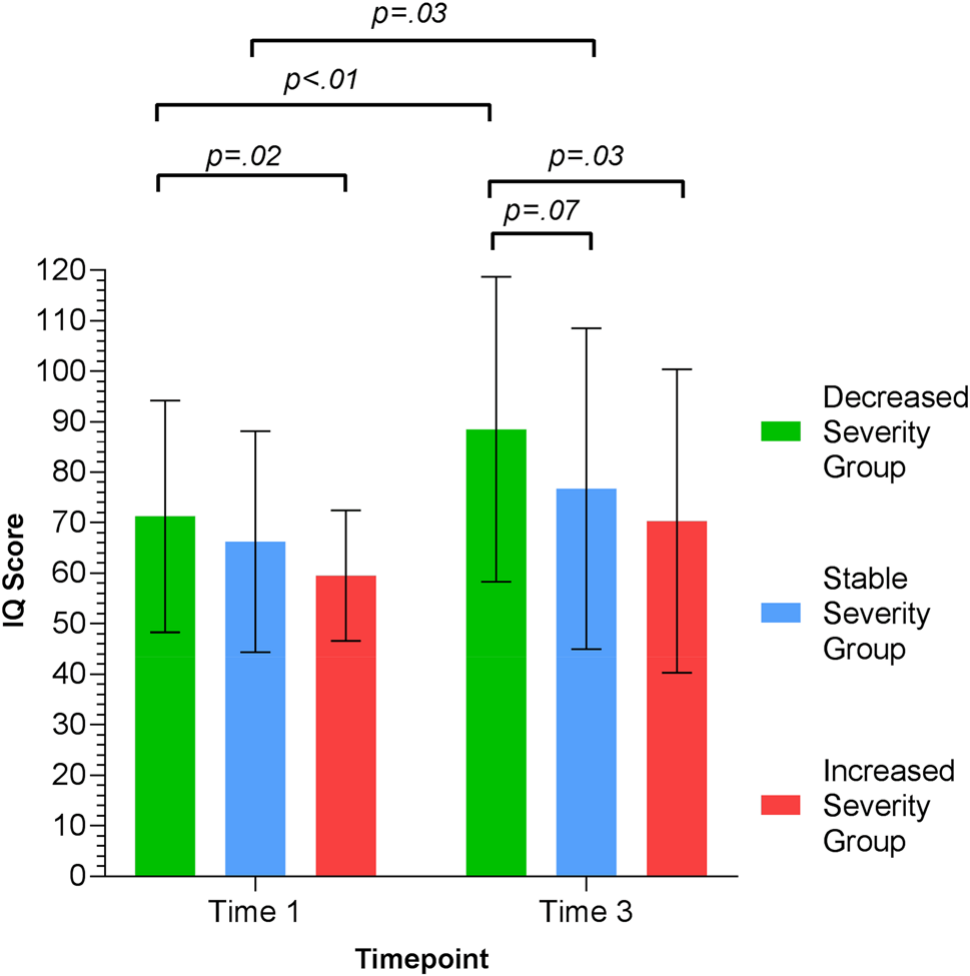
Mean IQ scores at Time 1 and Time 3 for the three groups. The DSG and SSG made substantial IQ gains over time. The DSGs’ mean IQ was higher than the ISG at both Time 1 and Time 3. The ISG remained stable in IQ over time

Group differences in IQ were examined at Time 1 and Time 3 (Table 2; Fig. 5). The DSG showed significant IQ gains over time (t(65.33) = − 2.72, *p* < 0.01, Cohen’s d = 0.64), as did the SSG (t(119) = − 2.24, *p* = 0.03, Cohen’s d = 0.38). This was not the case for the ISG (t(27.16) = − 1.52, *p* = 0.14). ANOVA of IQ by group across time showed significant differences in IQ between the groups (F(2,246) = 4.36, *p* = 0.01, Eta squared = 0.04). The DSG demonstrated overall (Time 1 and Time 3 combined) higher IQ than the ISG (*p* = 0.01) and trend level compared to the SSG (*p* = 0.09); the ISG and SSG did not differ (*p* = 0.35). Analysis of time points separately showed that the DSG had a higher mean IQ than the ISG at both Time 1 (t(54.97) = − 2.47, *p* = 0.02, Cohen’s d = 0.59) and Time 3 (t(42.16) = − 2.2, *p* = 0.03, Cohen’s d = 0.60).

### Adaptive Functioning for the Three Groups

**Fig. 6:**
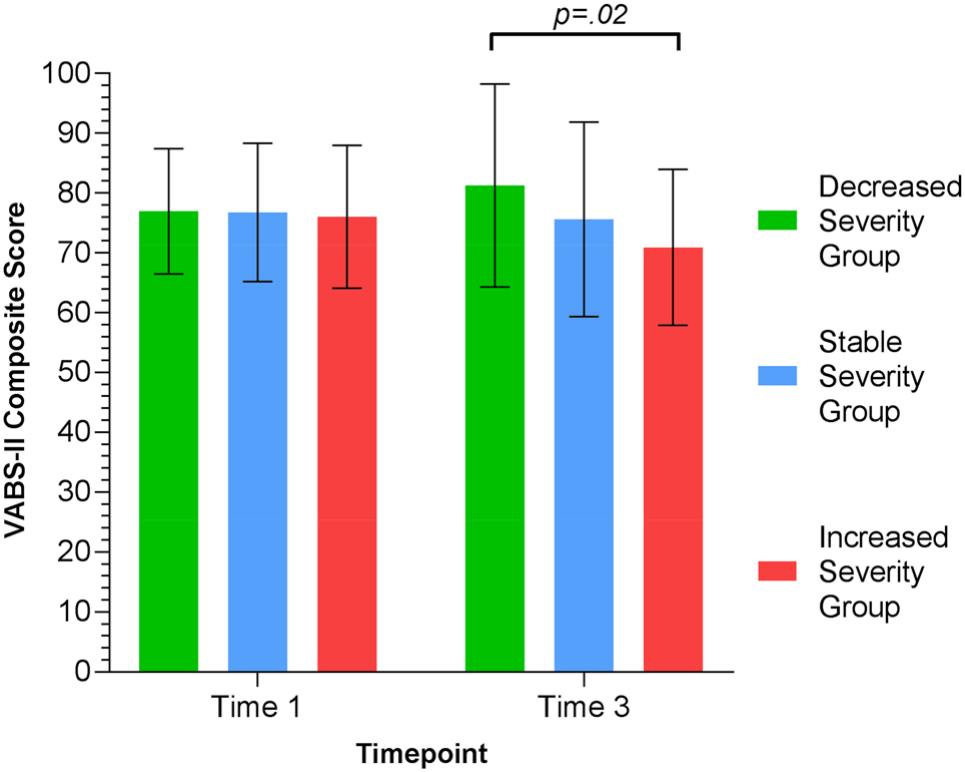
Mean adaptive function (VABS-II composite score) at Time 1 and Time 3 for the three groups. There were no differences between the groups at Time 1. At Time 3, the DSG had a higher adaptive function score compared with the ISG

Adaptive functioning differences were examined mainly using the VABS-II composite score as well as the domains scores (Table 2; Fig. 6). At Time 1, all groups had similar levels of adaptive functioning (F[2,114] = 0.04, *p* = 0.96). At time 3, the DSG had higher adaptive functioning than the ISG (t(34.35) = − 2.34, *p* = 0.02, Cohen’s d = 0.65). The SSG did not differ from the DSG (t(67.9) = 1.68, *p* = 0.1) or the ISG (t(25.58) = − 1.13, *p* = 0.27). The DSG was the only group to make gains in Communication (t(59.82) = − 2.43, *p* = 0.02, Cohen’s d = 0.58); its mean score at Time 3 was higher than the ISG (t(30.6) = 2.18, *p* = 0.04, Cohen’s d = 0.63). This group also showed higher Daily Living Skills at Time 3 compared to the SSG (t(68.77) = 2.19, *p* = 0.03, Cohen’s d = 0.47) and trend level improvements compared to the ISG (*p* = 0.06, Cohen’s d = 0.60). This was also the only group that did not experience a decrease in Motor Skills over time (t(63.46) = 0.24, *p* = 0.81).

### Optimal Outcome

A total of seven participants, 5.6% of the sample, had an ADOS CSS below the ASD cutoff at Time 3, thus potentially demonstrating optimal outcome. Six of these children were in the DSG (four girls and two boys) and one boy was in the SSG. These children had a mean severity level of 5 at Time 1 (range 4–7) and 1.8 at Time 3 (range 1–3). Their mean severity change was − 3.1 (range − 1 to − 6). All showed an increase in IQ over time, with IQ rising from a mean of 85.8 (range 75–95.8) to a mean of 105.3 (range 91–115). Adaptive functioning change (using the VABS-II composite score) was less consistent, as two children showed decreases and four showed increases over time (one child did not have a score at Time 1). Mean Time 1 adaptive function was 79.3 (range 71–92) and mean Time 3 was 89.6 (range 71–122).

## Discussion

The goal of the current study was to examine trajectories of autism symptom severity change in a rigorously diagnosed and recently ascertained cohort of autistic children between 3 and 6 years of age. Change scores were analyzed based on the Reliable Change Index and yielded three groups of different trajectories of autism symptom severity. A Decreased Severity Group (DSG) included children who decreased by 2 or more ADOS CSS points and comprised 28.8% of the total sample. This group was characterized by a large range of individual severity scores at Time 1, was over-represented with girls, had higher mean IQ at both time points and higher adaptive functioning at Time 3. The Stable Severity Group (SSG) included children with a change score of 1 point or less and comprised 54.4% of study participants. This group had an equal proportion of boys and girls, made IQ gains over time but remained stable in adaptive functioning. The Increased Severity Group (ISG) was comprised of participants who increased in severity by at least 2 points and accounted for 16.8% of the participants. Surprisingly, this group had the lowest mean severity score at Time 1 but the highest at Time 3. Girls were under-represented in this group and it showed lower and stable IQ and adaptive function scores over time. There were no significant differences in intervention intensity between the three groups.

### Comparison of Findings with Previous Studies

The amount and direction of change in autism severity described in previous studies has not been consistent. The current study demonstrates both similarities and differences with previous publications. In the earliest study using the ADOS CSS, Gotham et al. (2012) found that over 80% of participants demonstrated stable severity, with small groups decreasing or increasing over time. Their findings were largely corroborated by Venker et al. (2014). Szatmari et al. (2015) also reported mostly stable severity (89%) with a small group of participants which decreased in severity. Kim et al. (2016) reported 84% stability and a small group that increased in severity. These ealier studies were the basis for the general conclusion that the severity of a individual's autism does not change much following diagnosis. However, more recent studies have challenged this prevailing view. Kim et al. (2018) reported that only 23% of their participants remained stable over time and 52% either increased or decreased in severity. Pellicano et al. (2019), reported that 42% of their sample remained stable while 58% experienced a reliable increase or decrease in severity over time. Clark et al. (2017) also reported that, on average, children in their sample experienced significant change in symptom severity over time. Our own findings are consistent with a greater amount of change. While about half (54.4%) of the children in the Autism Phenome Project showed stability over time, 45.6% showed significant change. The potential for greater change of autism severity actually has a fairly long history from studies using a variety of measurement tools for symptoms (McGovern and Sigman 2005; Shattuck et al. 2007; Fountain et al. 2012; Gillespie-Lynch et al. 2012; Gulsrud et al. 2014; Barbaro and Dissanayake 2017; Hudry et al. 2018; Bal et al. 2019).

The direction of autism severity change has not been consistent in previous studies. Most previous studies reported some decrease in severity but the percentage of participants varied from 7–14% (Gotham et al. 2012; Venker et al. 2014; Szatmari et al. 2015) in earlier studies to 25–29% in more recent studies (Kim et al. 2018; Pellicano et al. 2019). Clark et al. (2017) indicate that, on average, children experienced a decrease in severity levels across early childhood. Consistent with these more recent studies, 28.8% of the participants in the current study decreased in severity. Significant decreases in autism symptom severity in young children has been demonstrated in several intervention studies either using a symptom-focused intervention (Pickles et al. 2016) or community-based interventions (Giserman-Kiss and Carter 2019).

Previous studies of severity have also identified individuals who increased in severity over time. Most previous studies indicate that 8–16% of their participants demonstrate a worsening trajectory (Gotham et al. 2012; Venker et al. 2014; Kim et al. 2016), while more recent studies report rates as high as 27–29% (Kim et al. 2018; Pellicano et al. 2019). The proportion of participants who increased in severity in the current study (16.8%) lies well within the range of those depicted in the past literature using the ADOS CSS.

### Sex Differences in Symptom Severity Change

We found that autistic girls decrease in severity more than boys and increase in severity less than boys during early childhood. These findings are somewhat at odds with the common notion that girls with autism are generally more impaired than boys (Lord et al. 1982; Carter et al. 2007). Yet, our results are consistent with many recent studies that suggest that girls might actually demonstrate better developmental outcomes than boys in the areas of cognition (Lai et al. 2012) sociability (Head et al. 2014), and pragmatic communication skills (Conlon et al. 2019). Mahendiran et al. (2019) showed that young girls diagnosed with ASD tend to show better social adaptive function compared to boys and Mandy et al. (2018) demonstrated that during early childhood girls show lower autistic social traits compared to boys. Infant sibling studies (6–12 months) have demonstrated that at-risk females show enhanced attention to social stimuli compared to both high-risk males and low risk males and females (Chawarska et al. 2016). Consistent with our findings, Szatmari et al. (2015) also found that girls were more likely to have less severe and decreasing symptoms, while boys were more likely to have more severe and stable symptoms. In fact, in a recent review of sex differences in the behavioral presentation of autism, Lai and Szatmari (2019) concluded that young autistic girls were more likely to have better cognitive development, less intense autistic symptoms and reduction of symptoms over time.

What could be leading to this sex difference? One possibility, as suggested by Lai et al. (2018), is that the social and cultural environments children grow up in impact girls and boys differently and may, in turn, influence brain function over the life span. For example, there is an expectation that girls participate in more social interactions compared to boys (Kreiser and White 2014; Bargiela et al. 2016). Parents have been shown to use more emotional references (such as emotion words) when talking with very young girls compared to boys (Aznar and Tenenbaum 2015). These sex differences emphasize girls’ socioemotional development from a young age (Chaplin and Aldao 2013) which might serve as “naturalistic interventions”, potentially supporting and leading to symptom severity decrease over time (Lai et al. 2018).

Another possibility relates to the increasingly accepted notion that girls and boys with autism might be characterized with different clinical presentations of symptoms (Frazier et al. 2014) which also develop differently across life (Mandy et al. 2018; Mahendiran et al. 2019). This presents a real challenge for current measurement instruments, as these sex-based behavioral differences might not be sufficiently captured by standard measures (Lai and Szatmari 2019). In a recent review, Lai and Szatmari (2019) characterized female autism to include female-gender-typical narrow interests, higher social attention, linguistic abilities, motivation for friendship and more camouflaging behaviors than autistic males. Camouflaging of autistic characteristics is a social compensatory behavior, or coping strategy, aimed at masking one’s symptoms in social situations (Hull et al. 2017). In an observational setting such as the ADOS, engaging in camouflage could lead to less severe scores as atypical social-communication features are masked from the assessor (Livingston and Happe 2017; Lai et al. 2018; Ratto et al. 2018). Camouflage has been shown to be more prevalent in females diagnosed with ASD compared to males across different age ranges, including adult women (Lai et al. 2017; Schuck et al. 2019), 10-year-old (Ratto et al. 2018) and 7–8-year-old (Dean et al. 2017) girls. Thus, the fact that more of the girls in this study appear to have decreased in autism severity based on the ADOS may actually be due to an increasing number of girls compared to boys who, with age, have learned how to mask their symptoms. We will explore this possibility in future studies.

### Is Initial Autism Severity a Predictor of Severity Change?

For most children who were participants in this study, their autism symptom severity level at age 3 was not a good predictor of the severity change they underwent during early childhood. We found that a large range of initial severity scores could lead to relative stability, decreasing severity or increasing severity. This is consistent with Pellicano et al. (2019) who found no association between initial severity level and the change an individual underwent across a 9 year period. Other studies have also failed to identify a relationship between early severity levels and future symptom change (Sutera et al. 2007; Bal et al. 2019). The children in the current study who remained stable or decreased in severity over time were characterized by large individual variation in severity levels at 3 years of age. Interestingly, the group of children who increased in severity showed significantly lower severity levels at age 3 and their severity scores were less variable than the other groups. Lower initial severity levels for groups that increase in severity over time were also observed in previous studies (Gotham et al. 2012; Venker et al. 2014; Kim et al. 2016, 2018).

### Is Intervention History Associated with Differences in Severity Change?

The large majority of children in the Autism Phenome Project and GAIN study have received substantial amounts of intervention across childhood. Analysis of intervention history (total number of hours of intervention received and intensity of intervention) did not show significant differences between the groups. These results are consistent with Gotham et al (2012) and Giserman-Kiss and Carter (2019), who found no association between intervention characteristics and severity change. Thus, it is unlikely that differences in symptom severity change are determined by differences in intervention history. That is not to say that there might be subtle differences between the groups in intervention experiences. For example, the children in the ISG had both the lowest symptom severity level at Time 1 and the lowest number of intervention hours received by Time 1 compared to the other groups. By Time 3, as they increased in symptom severity, their mean number of intervention hours had increased and was no longer the lowest of the groups. At Time 3, it was the DSG that had the lowest mean number of intervention hours up to that point and their intervention intensity had decreased compared to Time 1 as well. Thus, for the DSG, as their symptom severity decreased so did the amount of intervention they received. A number of studies have shown that children with lower symptom severity levels receive less or less intensive intervention (White et al. 2007; Anderson et al. 2009; Wei et al. 2014; Kim et al. 2018). Our observations are consistent with this.

### Is IQ Associated with Differences in Severity Change?

IQ demonstrated a significant, negative relationship with symptom severity change; as IQ scores increased from age 3 to age 6, symptom severity levels decreased. While both the DSG and SSG made IQ gains over time, the ISG did not. The DSG also had higher IQ compared to the ISG at both time points and compared to the SSG at Time 3. Findings that those with higher IQs were more likely to show a reduction in ASD symptoms is consistent with previous results in the APP cohort (Solomon et al. 2018). Gotham et al. (2012) also reported verbal IQ (VIQ) was a significant predictor of severity group membership. Children who decreased in symptoms were initially higher in VIQ, made the greatest gains and had the highest VIQ scores at age 6. IQ is considered to be the strongest predictor of outcomes for individuals with ASD (Volkmar 2002; Howlin et al. 2004). The current study’s results support this, showing that children who decreased in severity had higher IQs and made greater gains over time.

### How is Adaptive Function Associated with Autism Severity Change?

Adaptive Functioning also demonstrated a significant, negative relationship with severity change. As symptom severity decreased from age 3 to age 6, adaptive functioning increased. While there were no differences between the groups in level of adaptive functioning at age 3, by age 6 the DSG had higher adaptive function scores compared to the ISG. The interdependence of autism symptom severity and adaptive functioning has been previously documented (Perry et al. 2009; Charman et al. 2011; Gotham et al. 2012), yet other studies (Szatmari et al. 2015; Kim et al. 2016; Pellicano et al. 2019) showed little overlap between symptom severity and adaptive functioning trajectories.

While previous studies have shown mixed results concerning the relationship between symptom severity change and adaptive functioning, we found that it was the DSG specifically who, in addition to declining in symptoms, demonstrated better adaptive skills in multiple domains compared to the other groups. This group increased in both the Communication and Daily Living Skills domains and was the only group not to have experienced a decline in Motor Skills domain. Both language development (Bavin et al. 2014) and non-verbal communication skills (Kjellmer et al. 2012; Lobban-Shymko et al. 2017), two areas within the communication domain, have previously been shown to associate with or predict autism symptom severity levels. Motor ability has also been demonstrated to be relevant to symptom severity; typical motor development at a young age is a predictor for optimal outcome (Helt et al. 2008), while delays in motor skills have been shown to be prevalent in the ASD population (Lloyd et al. 2013).

### Optimal Outcome and Severity Change over Time

This study was initially motivated by the phenomenon of optimal outcome. Optimal outcome is traditionally defined as a decrease in autism symptoms in individuals previously diagnosed with ASD, so that they no longer meet diagnostic criteria (Fein et al. 2013). A total of seven participants, 5.6% of our sample, received an ADOS CSS below the ASD cutoff (1–3) at Time 3. Six of these children were in the DSG (four girls and two boys) and one boy was in the SSG. Since Optimal outcome is defined based on different aspects of function as well as autism symptom level (Fein et al. 2013), additional evaluations would have to be carried out concerning both the home and educational environments to confirm that these children have actually achieved optimal outcome.

Optimal outcome might also be interpreted more generally as indicating significant intra-individual change rather than the attainment of a specific cut-off score. This definition takes a wider approach to understanding the complex and variable ways in which children with autism grow and develop (Georgiades and Kasari 2018). If we apply this perspective to the current study’s results, the notion of optimal outcome would be relevant to many more children in the DSG who, while not decreasing below the ASD cut-off score, experienced substantial personal decrease in autism severity over time.

## Limitations

This study had some limiting factors. First, the sample size of 125 participants is modest compared to the size of the samples used in some of the previous reports. However, this sample incorporates participants with a wide range of severity, cognitive and function levels. Moreover, the clinical assessment and cognitive testing is rigorously carried out at one site and administered by experts in child development. Second, the current study is based on two early childhood time points. We hope to gather further longitudinal information in the future and to extend these findings in time. Third, the change in autism severity is based only on the calibrated severity score of the ADOS. It would be valuable to employ other objective measures of autism severity to confirm our findings and to explore potentially "artifactual" decreases in autism severity that may result from sex differences in symptom manifestation across time. Fourth, the current study raises several important issues which require further investigation, such as the relationships between IQ, initial severity level, and type and intensity of intervention received, in relation to symptom change over time.

## Implications

Studies of autism severity change are of particular interest to parents and clinicians alike. There is good news in the current study that nearly 30% of young children have less severe autism symptoms at 6 than they did at 3; some even lose their diagnosis entirely. We do not currently know how to predict with certainty which children will follow this positive trajectory. Somewhat more disheartening is the finding that a sizable group of children will experience a worsening of autism symptoms following diagnosis. Again, it is not possible to predict who these children are so that they might receive added intervention. An emerging literature indicates that there are a variety of risk factors related to outcomes, and also points out the need to gain a better understanding of protective factors (Elsabbagh 2020). Prospective studies focusing on at-risk populations for ASD (infant sibling studies) have shown that a regressive onset of symptoms might be the rule rather than the exception (Ozonoff and Iosif 2019). Most toddlers diagnosed with ASD seem to lose social-communication abilities that had already been acquired in infancy, prior to the development of autism symptoms. Thus, it is possible that the increased severity group is showing an extension of this regressive course into early childhood. Longitudinal studies of larger groups of participants that combine both intensive behavioral as well as biological assessments may ultimately define biomarkers that are better able to assign a child to one of the severity trajectory groups. This would be an important first step to promoting decreases and reducing increases in autism severity over time. Several intervention methods have demonstrated the ability to impact symptom severity levels, each utilizing a different therapeutic approach. These include the Early Start Denver Model (Dawson et al. 2012; Estes et al. 2015), Neurofeedback and Biofeedback (Goodman et al. 2018), parent-mediated social communication therapy (PACT) (Pickles et al. 2016; Torjesen 2016) and the Early Social Interaction (ESI) model (Wetherby et al. 2018). There is a growing emphasis on identifying specific predictors of symptom change in order to “match” interventions with child characteristics (Hudry et al. 2018). In this regard, it would be helpful to identify which type of approach would be most beneficial for the developmental profiles of children who either increase, decrease or remain stable in severity across early childhood.

## Conclusions

This study is consistent with a growing literature that indicates that there is the potential for substantial change in autism symptom severity over time. Because the current study had a higher proportion of girls than previous studies, it became evident that girls tend to decrease more and increase less in autism severity than boys over time. The reason(s) for this sex difference need further exploration. The current study focused on overall autism severity and did not attempt to break down severity into its social communication and repetitive behavior components. We plan to explore this issue as well and to extend in time the trajectory of autism severity as the participants of the Autism Phenome Project enter middle childhood and adolescence. We appreciate that this work will be relevant for families, professionals and researchers as it establishes expectations for long term outcome once a diagnosis is obtained. “Tailoring” intervention according to a child’s prognosis and needs could support future severity decreases and attempt to prevent severity increases, in order to maximize the potential of each child.
